# Acute Large Bowel Obstruction Post Umbilical Hernia Repair Surgery: A Case Report

**DOI:** 10.7759/cureus.72566

**Published:** 2024-10-28

**Authors:** Nguyen Dang, Selwyn Kay

**Affiliations:** 1 Osteopathic Medicine, California Health Sciences University, Clovis, USA; 2 Surgery, Bakersfield Memorial, Bakersfield, USA

**Keywords:** closed loop obstruction, diverticular abscess, diverticulitis, large-bowel obstruction, sigmoid diverticulitis, umbilical hernia repair

## Abstract

While large bowel obstruction (LBO) is a less common cause of bowel obstruction, it has a broad range of differentials. Its most common etiology in the United States is colorectal adenocarcinoma. Patients usually present with diffuse abdominal pain, constipation, abdominal distension, and nausea. Abdominal X-rays can quickly assess bowel dilation, but a CT scan is the gold standard to determine the location and severity of the blockage. The treatment can range from IV fluid to emergent colectomy.

A 41-year-old male with a history of bronchitis and hypertension presented for a surgical consult six days post-incarcerated umbilical hernia repair. The patient complained of nausea, diffuse abdominal distension and tenderness, and inability to pass gas. Physical examination revealed a severely distended abdomen with rebound and guarding at the lower right quadrant. CT scan showed severely dilated ascending colon and cecum along with pneumatosis. There was also an obstructing mass at the sigmoid colon. A diagnosis of severe LBO with impending perforation was made, and the patient underwent emergent exploratory laparotomy. Subtotal colectomy was done with anastomosis between the ileum and sigmoid colon. The pathology report showed an 8.0 cm dilated cecum, necrosis of the ascending colon, and diverticulitis with scar fibrosis along the wall of the colonic membrane. The patient had an ileus and kidney injury postoperatively but they subsequently resolved.

Diverticulitis is historically common in the elderly population, but recent studies have shown a rising incidence among younger individuals. It is a common cause of obstruction but rarely causes severe complications like pneumatosis and perforation that require emergent surgery. When occluded, the mass and patent ileocecal valve can cause closed-loop bowel syndrome. Even though an unhealthy diet is a known trigger for diverticulitis, surgery is possibly another factor in rare cases. Patients of all ages should be educated on the symptoms of diverticular disease, and surgical monitoring is key in those with known diverticulosis.

## Introduction

Large bowel obstruction (LBO) is a less common cause of bowel obstruction that requires surgical interventions. The differentials in these cases can be classified into three categories: lumen (fecal impaction), intrinsic, and extrinsic. The most common intrinsic cause in the United States is colorectal adenocarcinoma, especially in the elderly population [1,2]. Other intrinsic causes include volvulus, diverticulosis, inflammatory bowel disease, intussusception, stricture, and other less common tumors. The extrinsic causes include adhesions from surgery, inguinal hernia, endometriosis, and external compression [1]. 

The usual symptoms of LBO are diffuse abdominal pain, constipation, abdominal distension, and nausea, which can be similar to small bowel obstruction [2]. Due to the prevalence of colorectal adenocarcinoma, it is important to inquire about the history of colorectal cancer in patients, as well as symptoms like gastrointestinal bleeding, unintentional weight loss, and changes in bowel habits. Imaging is required to determine the severity and etiology of the condition. The abdominal plain film helps to assess the large bowel dilation quickly. An abdominal CT scan with IV contrast is the gold standard to determine the location of the blockage as well as its etiology [1,2]. Depending on the severity and destruction of the large bowel, treatments can range from nonoperative approaches, like IV fluid and antibiotics, to operative methods, like endoscopy and colectomy during explorative laparotomy. Emergent surgery is indicated if there are signs of abscess, peritonitis, pneumatosis, and perforation [1]. 

This article was accepted as a virtual poster presentation on April 12, 2024, at the fifth Edition of the Global Conference on Surgery and Anaesthesia and will be presented on September 5-7, 2024.

## Case presentation

**Figure 1:**
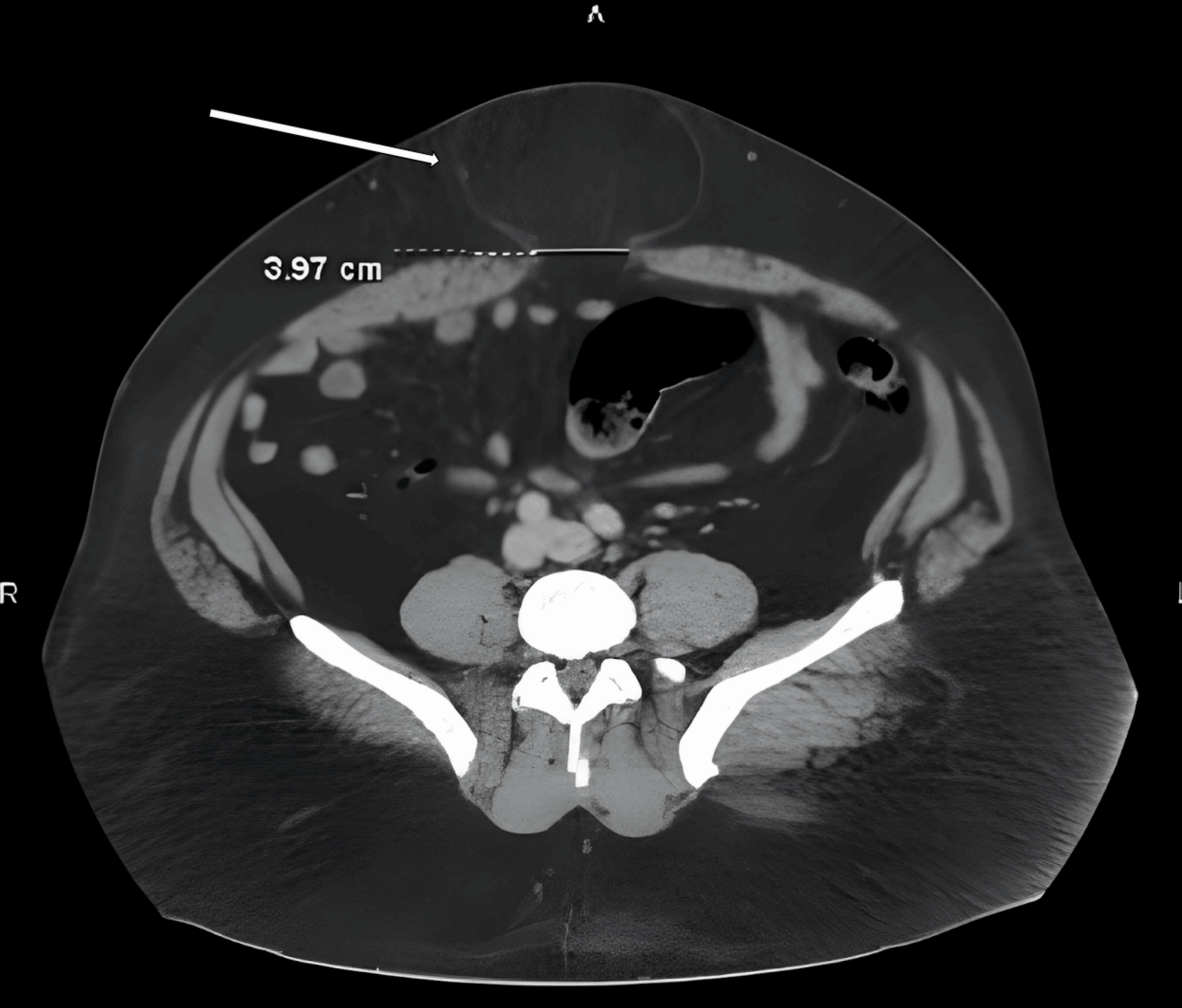
Transverse CT scan before hernia surgery showing hernia defect with the protruding hernia sac (white arrow) CT: computed tomography

The patient was a 41-year-old male who had undergone umbilical hernia repair by a different surgical team six days ago. He had been initially admitted due to a painful abdominal mass with constipation. His past medical history included bronchitis and hypertension managed with albuterol, budesonide inhalers, and amlodipine. He had a BMI of 39.2 kg/m^2^ and denied any vomiting, fevers, chills, previous abdominal surgeries, or colonoscopies. CT scan then showed a 3.97 x 1.8 cm abdominal hernia with a large fat protrusion, as well as moderate diverticulosis at the descending colon without obstruction (Figure 1). A diagnosis of acute exacerbation of a chronically incarcerated umbilical hernia had been made. Open hernia repair had been performed, showing a 4.0 cm fascial defect. The hernia sac had been taken down, and the contents had been revealed to be viable omentum. The hernia had been closed and reinforced with a mesh overlay. No intraoperative complications had been reported.

**Figure 2:**
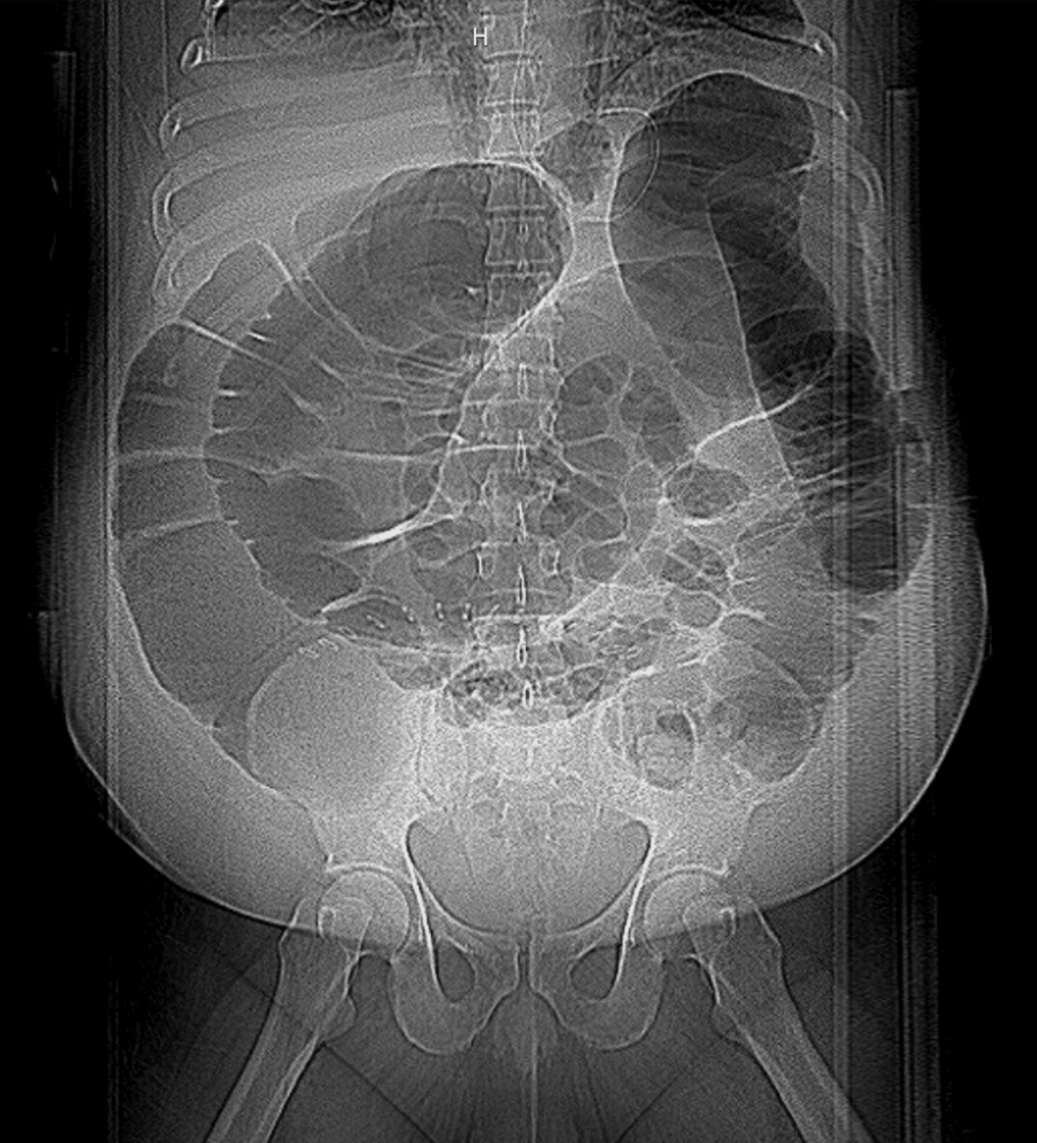
Abdominal X-ray showing dilated large intestine

Over the next six days, the patient had continued to have nausea, a distended abdomen without worsening, and an inability to pass gas. IV piperacillin-tazobactam and vancomycin had been started on postoperative day (POD) one. However, white blood count (WBC) continued to increase from 11.5 thousand/μL on POD one to 13.6 thousand/μL on POD six. On POD seven, the patient complained of worsening abdominal discomfort. Physical exam showed diffusely distended and tympanic abdomen with significant rebound and guarding at the lower right quadrant. WBC increased to 18.8 thousand/μL. X-ray showed severe dilation of the large intestine, suggesting ileus (Figure 2).

**Figure 3:**
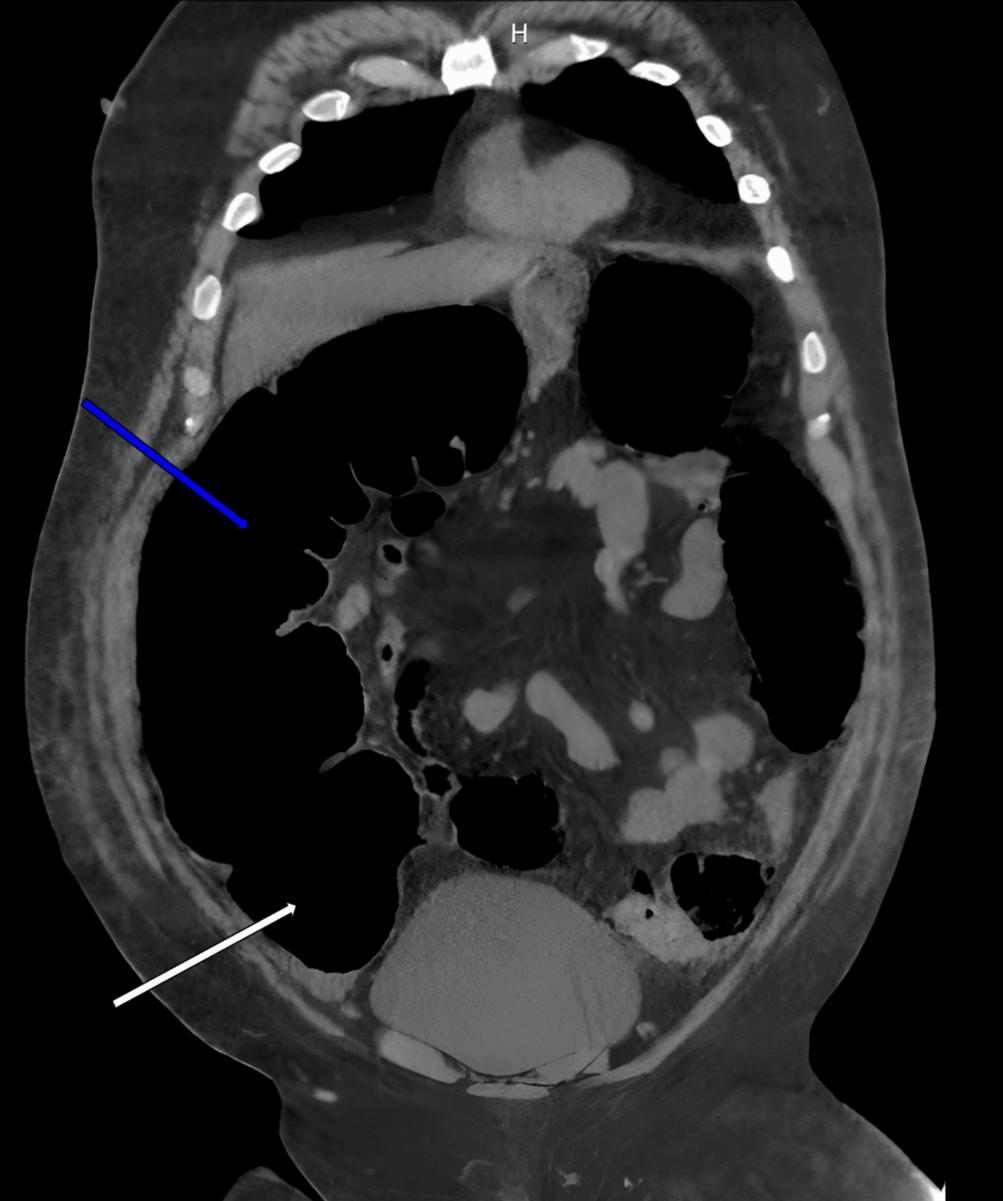
Coronal CT scan before colectomy showing dilated cecum (white arrow) and ascending colon (blue arrow) CT: computed tomography

**Figure 4:**
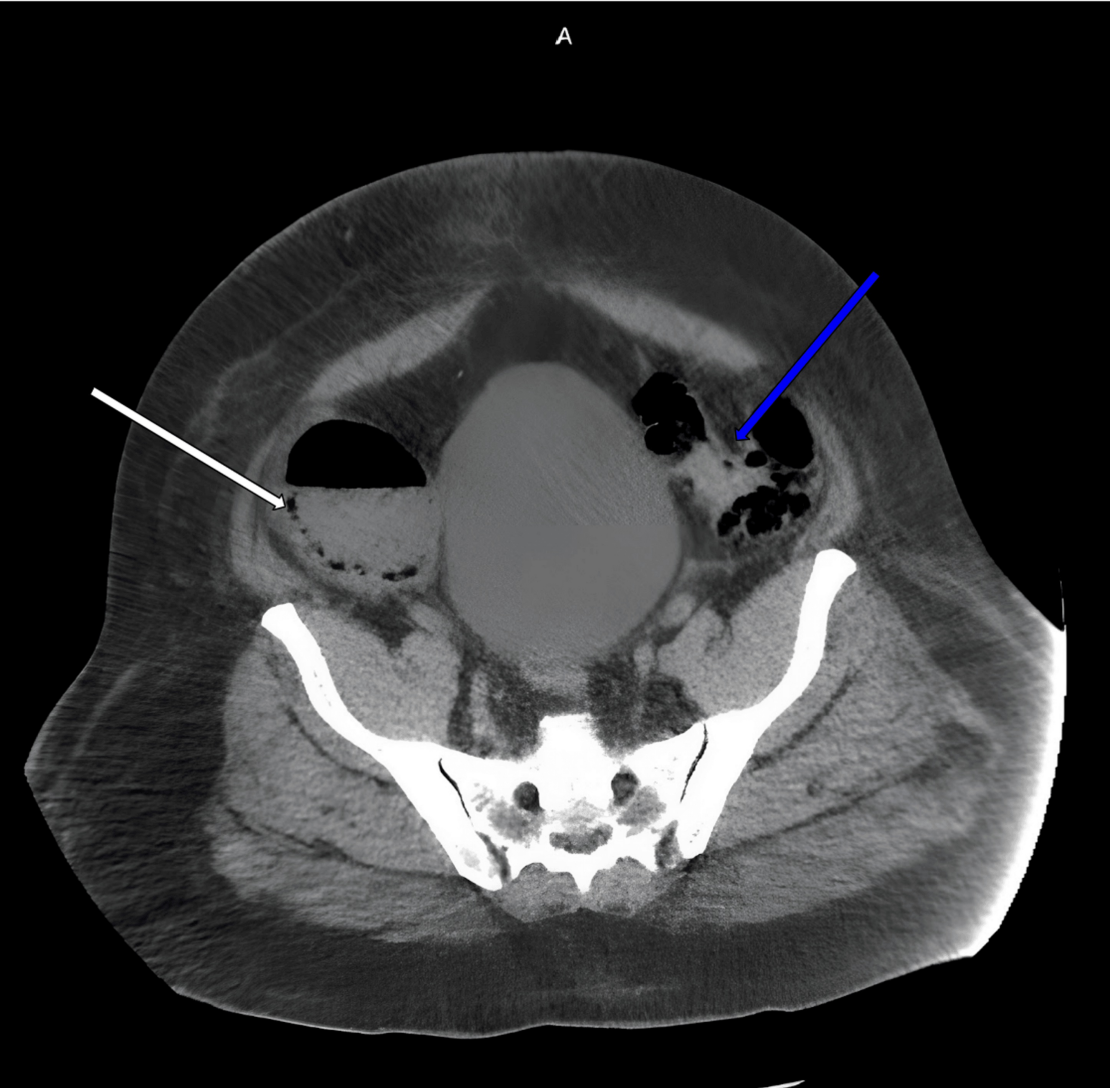
Transverse CT scan before colectomy showing pneumatosis at the ascending colon (white arrow) and the obstructing mass at the sigmoid colon (blue arrow) CT: computed tomography

CT scan of the abdomen showed moderate to severely distended cecum, transverse, and portions of the descending colon (Figures 3-4). The distension was most significant at the cecum and ascending colon. There was also pneumatosis at the wall of the cecum and ascending colon. At the sigmoid colon, there was a possible mass causing obstruction. A diagnosis of severe LBO with impending perforation was considered. Nonoperative options, including observation with continued nasogastric tube and IV antibiotics, and operative options were discussed extensively with the patient. We emphasized the severity of the LBO with its impending complication, and the patient opted for an emergent exploratory laparotomy with possible bowel resection and colectomy.

**Figure 5:**
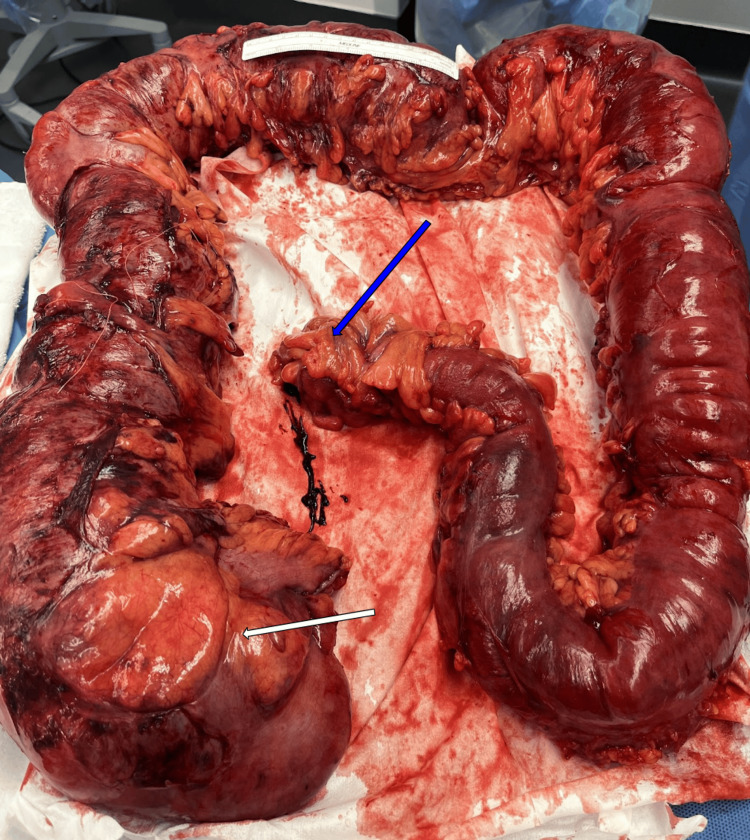
Resected large intestine showing severely dilated cecum (white arrow). The sigmoid colon was resected at the area distal to the obstructing mass (blue arrow).

We encountered a massively dilated colon during the procedure, extending from the ileocecal valve to the sigmoid colon. At the sigmoid colon, there was a palpable obstructing mass without any evidence of extension into the pelvic sidewall, lymph nodes, or any sign of metastatic disease. The decision was made to perform a subtotal colectomy. The portion from the distal end of the terminal ileum to the spot distal to the obstructing mass of the sigmoid colon was carefully removed (Figure 5), followed by end-to-end anastomosis.

Pathological analysis of the resected colon was performed. The cecum lumen was dilated to a diameter of 8.0 cm. The ascending colon lumen had extensively thinned, and the mucosal surface showed necrosis, hemorrhage, and ulceration. Sectioning of the mass at the distal sigmoid colon showed diverticulitis with abscess formation, perforation, and associated scar fibrosis. There were multiple pericolonic lymph nodes without evidence of malignancy.

During the postoperative period, the patient developed an ileus and acute kidney injury, which subsequently resolved. The patient also had an infection at the surgical site with a negative culture. He still required vacuum-assisted closure (VAC) for the wound and was discharged home on day 15 with wound VAC and antibiotics.

## Discussion

An occlusion is one of the most severe complications of LBO. With a closed ileocecal valve, the segment of the large intestine from the valve to the occlusion site becomes a closed loop [1,2]. As pressure builds up in the large intestine, the lumen will slowly or rapidly expand, causing a severely distended large intestine. As it is a transition point, the sigmoid colon is the most common occluded site [3]. This usually leads to distension of the preceding descending and ascending colon. However, the cecum is the site with the most distension. Laplace's law states that the area with the smallest radius will experience the most pressure. As the large intestine expands, the wall becomes compromised, causing pneumatosis and eventually perforation. Our patient's presentation was consistent with this pathophysiology. He had nausea, obstipation, and abdominal distention for six days, which significantly worsened on day seven. CT scan showed air in the colonic wall, consistent with pneumatosis intestinalis and impending perforation. Postop pathology showed a severely dilated cecum, a distended and damaged ascending colon, and an occluding sigmoid mass.

Furthermore, the mass was determined to be an abscess caused by diverticulitis, the second most common cause of LBO in the United States [1]. Historically, diverticulosis and diverticulitis are far more common in patients aged over 60 years [4]. However, several studies have shown a rising incidence of diverticulitis in younger patients (<50 years) [5]. These studies do not suggest that younger age is a risk factor for more severe complications like obstruction and perforation [4]. A large population-based study in the United Kingdom has shown that colonic perforation from diverticular disease incidence is about 2.66 per 100,000 person-years in patients aged under 45 years [6]. The study also reported an increased incidence of perforation over 18 years. These complications occur due to repeat episodes of diverticulitis, causing fibrosis, abscess, and compression of the colonic lumen [1]. Hence, the risk of stricture and obstruction is higher with older age. However, the incidence of emergent surgery is only two per 100,000 person-years [4]. Although our patient did not provide a history of diverticulitis, the CT scan results were consistent with chronic and recurrent diverticulitis. The insidious onset and deterioration fit the presentation of closed-loop bowel syndrome, and the patient's age and need for emergent surgery made the case rare and alarming.

Moreover, the diverticulitis had started after the uneventful umbilical hernia surgery, making the case interesting. It is possible that the incarcerated umbilical hernia surgery was the trigger for the acute diverticulitis episode. Even though the pathophysiology is not entirely understood, the two leading theories for the cause of diverticulitis are chronic inflammation and alteration in the gut microbiome [5]. This is supported by the fact that diabetes, obesity, smoking, and low-fiber diets are risk factors that can change the microbiome environment, causing inflammation of the mucosa. Therefore, even though there were no postop laboratory values that showed the extent of inflammation, it is possible that the surgical stress put the patient in an inflammatory state, leading to the acute exasperation of diverticulitis [7].

A retrospective cohort study tackled this rare phenomenon by recruiting patients who went into the clinic for colonoscopy and looking at the history of prior abdominal surgeries and the incidence of diverticulosis and diverticulitis after [8]. The study only found a statistically significant association between inguinal hernia repair and the emergence of diverticulosis and diverticulitis. The authors hypothesized that adhesions increased abdominal pressure, which led to diverticulosis, but concluded that other unknown mechanisms could be responsible instead. Another retrospective cohort study with a similar method found an association between direct inguinal hernia and umbilical hernia repair and diverticulosis [9]. Although neither study provided a working mechanism, the association is still possible in our patient, given that the umbilical hernia surgery had no other complications.

Overall, due to the current rise in the incidence of diverticulosis and diverticulitis in young adults, educating patients on managing this common disease is essential. Since diet is one of the most significant risk factors, physicians should advise patients to maintain a diet high in fiber, including fruits and vegetables [10]. Patients should be made aware of the signs of diverticulitis - fever, worsening abdominal pain despite medications, and inability to tolerate fluids - and advised to seek medical attention if they experience them. Diverticulosis is mainly a clinical diagnosis, but a CT scan is the definitive diagnostic modality. The surgical team needs to monitor patients closely if diverticulosis is seen on imaging before surgery to prevent possible acute diverticulitis.

## Conclusions

The incidences of diverticulosis and diverticulitis are on the rise in younger population. Diverticulitis is one of the most common causes of bowel obstruction in the United States, and its recurrence can lead to severe complications such as abscess and perforation. Patients with diet-related concerns or a history of diverticular disease at any age should be educated on recognizing symptoms of diverticulitis and developing healthy lifestyles to prevent the onset or recurrence of diverticulitis. Recent surgery is a possible trigger of diverticulitis, as it can cause transient stress on the body, and hence high-risk patients require close preop and postop monitoring.
